# The most important tasks for peer reviewers evaluating a randomized controlled trial are not congruent with the tasks most often requested by journal editors

**DOI:** 10.1186/s12916-015-0395-3

**Published:** 2015-07-03

**Authors:** Anthony Chauvin, Philippe Ravaud, Gabriel Baron, Caroline Barnes, Isabelle Boutron

**Affiliations:** Centre d’Épidémiologie Clinique, Hôpital Hôtel Dieu, Assistance Publique des Hôpitaux de Paris, Paris, France; Paris Descartes University, Paris, France; INSERM, UMR 1153, Centre of Research in Epidemiology and Statistics Sorbonne Paris Cité – (CRESS), METHODS team, Paris, France

**Keywords:** Peer review, Q-sort, Randomized controlled trials, Recommendations to reviewers

## Abstract

**Background:**

The peer review process is a cornerstone of biomedical research publications. However, it may fail to allow the publication of high-quality articles. We aimed to identify and sort, according to their importance, all tasks that are expected from peer reviewers when evaluating a manuscript reporting the results of a randomized controlled trial (RCT) and to determine which of these tasks are clearly requested by editors in their recommendations to peer reviewers.

**Methods:**

We identified the tasks expected of peer reviewers from 1) a systematic review of the published literature and 2) recommendations to peer reviewers for 171 journals (i.e., 10 journals with the highest impact factor for 14 different medical areas and all journals indexed in PubMed that published more than 15 RCTs over 3 months regardless of the medical area). Participants who had peer-reviewed at least one report of an RCT had to classify the importance of each task relative to other tasks using a Q-sort technique. Finally, we evaluated editors’ recommendations to authors to determine which tasks were clearly requested by editors in their recommendations to peer reviewers.

**Results:**

The Q-sort survey was completed by 203 participants, 93 (46 %) with clinical expertise, 72 (36 %) with methodological/statistical expertise, 17 (8 %) with expertise in both areas, and 21 (10 %) with other expertise. The task rated most important by participants (evaluating the risk of bias) was clearly requested by only 5 % of editors. In contrast, the task most frequently requested by editors (provide recommendations for publication), was rated in the first tertile only by 21 % of all participants.

**Conclusions:**

The most important tasks for peer reviewers were not congruent with the tasks most often requested by journal editors in their guidelines to reviewers.

**Electronic supplementary material:**

The online version of this article (doi:10.1186/s12916-015-0395-3) contains supplementary material, which is available to authorized users.

## Background

The peer review process is a cornerstone of biomedical research publication [1]. The editorial peer review process is described as journal editors relying on the views of independent experts in making decisions on, for example, the publication of submitted manuscripts or presentation of reports at meetings [2]. The peer review system is considered the best method for evaluating publications of health research [3]. Nevertheless, the effectiveness of the peer review system is questioned. In 2010, a report ordered by the UK House of Commons showed that the cost to the UK Higher Education Institutions in terms of staff time was £110 to £165 million per year for peer review and up to £30 million per year for the work done by editors and editorial boards [4]. Worldwide, peer review costs an estimated £1.9 billion annually and accounts for about one-quarter of the overall costs of scholarly publishing and distribution [5]. The human cost was evaluated at 15 million hours by 2010 [6]. Furthermore, the peer review system may fail to identify important flaws in the quality of manuscripts and published articles [7, 8]. A recent study by Hopewell et al. [9] showed that peer reviewers could not detect important deficiencies in the reporting of methods and results of randomized trials; modifications requested by peer reviewers were relatively minor. For example, reviewers added information about concealment of allocation sequence for 10 % of the manuscripts while it was not reported in more than half of the articles. Although most had a positive impact, some were inappropriate and could have a negative impact on reporting in the final publication. Peer reviewers’ assessments can also be affected by the study results: peer reviewers tend to rate the methodological quality of a study with positive results higher than the same study reported with negative results [10, 11]. Moreover, the reproducibility between reviewers is disputable [12–14]. Finally, peer reviewers are not able to detect fraud [15] and mistakes [16].

Our hypothesis is that several tasks can be expected from peer reviewers and that peer reviewers and editors may have different views on which task is important.

We aimed to 1) identify all tasks that are expected of peer reviewers when evaluating a manuscript reporting a randomized controlled trial (RCT), 2) sort these tasks according to their importance, and 3) determine which tasks are clearly requested by editors in their recommendations to reviewers. We focused on the peer review of RCTs because this design is the cornerstone of therapeutic evaluation.

## Methods

We proceeded in three steps: first, we identified and combined the tasks expected of peer reviewers, then surveyed peer reviewers of RCT reports to sort the different combined tasks, followed by assessment of the editors’ recommendations to peer reviewers to determine which of the tasks identified are clearly requested.

### Identification of tasks expected from peer reviewers

To identify the tasks expected of peer reviewers, we systematically reviewed the published literature and searched for the recommendations to reviewers in a sample of journals.

### Systematic review of the published literature

We systematically searched MEDLINE via PubMed, Google Scholar, and EMBASE for all articles related to the peer review process and reporting tasks expected of peer reviewers published in English during the last 10 years with an abstract available. The search strategy used the keywords “*peer review*” OR “*peer-review*” (Search date: March 18, 2014). One researcher (AC) read all titles and abstracts and all text fragments from full-text articles. If a study was potentially eligible, the full-text article was retrieved. We included articles in the field of biomedical research dedicated to the peer review process and specifically pertaining to tasks expected of peer reviewers.

### Journal recommendations to reviewers

We selected a sample of journals publishing full-text articles in English. We aimed to select a large variety of journals publishing RCT results. For this purpose, we proceeded in two steps. 1) We searched PubMed with the keywords *randomized controlled trial* with the following filters: study type RCT, English language, human studies, published between January 1 and March 31, 2013, and with an abstract available (search date February 2, 2014). All citations retrieved were exported to an EndNote file. We selected all journals whatever the category (Additional file 1) that had published at least 15 reports of RCTs indexed in PubMed during the 3-month period. 2) We arbitrarily selected, in the Web of Science (Journal Citation Reports), 14 categories in the field of medicine among the 111 existing categories in the field of (bio)medicine. We chose these categories to have a sample of journals that focused on a large variety of patients (children, adults) and treatments (drugs, surgery, rehabilitation, psychotherapy, radiotherapy, devices, etc.).The selected categories were emergency medicine, surgery, internal medicine, anesthesiology, cardiac and vascular disease, critical care medicine, hematology, infectious diseases, neurosciences, oncology, pediatrics, psychiatry, rheumatology, and clinical neurology. Then, for each category, we selected the 10 highest-impact-factor journals (Journal Citation Report 2012) that published RCTs (Additional file 1). For this purpose, we hand-searched the three last issues of each journal (2014/04/30) and selected only journals that had published at least one RCT report in the three last issues.

For each selected journal, the recommendations to peer reviewers posted on the journal website were searched (by AC) and all the editors and managing editors with an available email address were emailed to ask for the recommendations they provide to their peer reviewers. When recommendations were provided on the journal website and the journal publisher website, we combined the recommendations and considered that the peer reviewers had to follow both recommendations. One reminder was sent every week until three emails were sent.

### Extraction and condensation of tasks

From all sources (articles, website, journal editors’ responses), one researcher (AC) systematically recorded all peer review tasks relevant for the assessment of an RCT reported. Duplicates were deleted and tasks were classified into the following categories used in a previous work [17]: 1) logistics (i.e., how to use the manuscript review system), 2) etiquette/objectives (i.e., how to behave during the peer review process), 3) abstract (i.e., form and content), 4) introduction (i.e., context of the study), 5) rationale (i.e., interest of the study), 6) methods (i.e., quality of study methods), 7) statistics (i.e., sample size calculation or proposition of a specific statistics review), 8) results (i.e., presentation and content of results), 9) discussion (i.e., interpretation of results and limitations), 10) conclusion (i.e., whether the conclusion is supported by results), 11) references (i.e., pertinence and presentation of references), 12) overall presentation (i.e., language, length and organization of the article), 13) figures/tables (i.e., presentation, size and number of figures), 14) reporting guidelines (i.e., reporting guidelines checklist such as CONSORT), 15) ethics (i.e., reference to ethics review board or respecting patient rights), and 16) fraud (i.e., risk of plagiarism or conflict of interest) (Additional file 2).

**Fig. 1:**
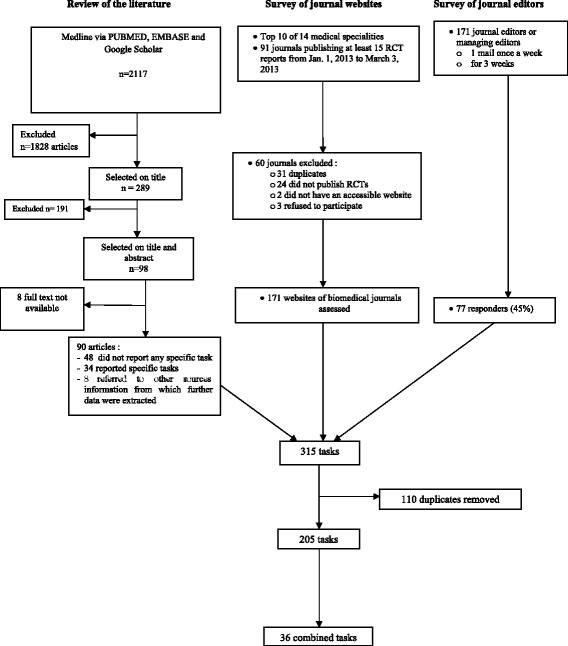
Flow chart of the survey of tasks expected of peer reviewers

All tasks were secondarily combined to create principal task combinations [18, 19] (Fig. 1). In fact, some of the tasks collected were defined precisely and were related to the same domain. For example, evaluating the risk of bias could imply several tasks: evaluating the quality of the randomization procedure, blinding of patients, care providers, outcome assessors, and rating and handling of missing data. Similarly, assessing the quality of randomization procedure can be divided into different tasks such as evaluating the allocation sequence generation and allocation concealment, which imply checking who generated the random allocation sequence, who enrolled participants, and who assigned participants to interventions and evaluating the mechanism used to implement the random allocation sequence (such as sequentially numbered containers), assessing any steps taken to conceal the sequence until interventions were assigned. We did not want to go into such details for defining a task, so we condensed all the tasks related to the assessment of the risk of bias into a single task. We proceeded similarly for all tasks that needed some condensation.

Two researchers (AC and CB) independently combined the tasks, and consensus was reached by discussion in case of disagreement. If no consensus was reached, a third researcher helped resolve any disagreements (IB).

### Survey of peer reviewers to sort the statements related to tasks by importance

To sort the tasks expected from peer reviewers, we used Q-sort [20, 21], which asks participants to sort combined tasks in relation to each other according to a predetermined distribution.

### Participants

We selected participants who had peer-reviewed at least one RCT report. Our aim was to obtain the participation of peer reviewers with different expertise (methodological and clinical expertise) and backgrounds. For this purpose we used a large strategy of recruitment and contacted the following participants:

- A convenience sample of participants listed as peer reviewers of the *New England Journal of Medicine*, *Journal of the American Medical Association*, and *Annals of the Rheumatic Diseases* cited by the journal in the last journal issues for 2010 and 2011 and selected in a previous study [22].
- Corresponding authors of 1) articles published in the 10 highest-impact-factor epidemiologic journals indexed in *Public, Environmental & Occupational Health* (Journal Citation Report 2012) and in the biostatistical medical journals *Statistics in Medicine, Biostatistics, Biometrics,* and *Statistical Methods in Medical Research* and 2) RCT articles indexed in PubMed between January 1 and June 15, 2014 (date of search: June 16, 2014 search strategy). For each article selected, we extracted the corresponding author’s email address, when available.
- Editors of the journals selected by searching journal websites described previously.

All participants were invited via a standardized and personalized email to participate in a survey to determine which tasks are most important when reviewing the report of an RCT. There was no reminder sent after the first email. We included only participants who reported in the survey that they had peer-reviewed at least one RCT report.

### Sorting tasks

With Q-sort, participants classify the importance of each task relative to other tasks [23]. We pilot-tested the survey with 10 researchers (two statisticians, six methodologists, and two clinicians) and improved the wording of some items. Sorting involved three steps. First, participants read a list of tasks identified and combined in a previous step that could be expected of a peer reviewer when evaluating the report of an RCT. Second, participants sorted the combined tasks into three categories according to their evaluation of the task’s importance: 1) less important, 2) neutral, or 3) more important. Third, participants were asked to read the sorted tasks and place them on a score sheet representing a normal distribution ranging from −5, less important, to +5, more important. An example of sorting is given in Additional file 3. Finally, participants could re-evaluate their distribution and shift items. We used FlashQ Software 1.0, the Flash application for performing Q-sort online [24].

### Assessment of tasks requested in editors’ recommendations to peer reviewers

For all tasks identified and sorted, we systematically determined whether the tasks were clearly recommended by journal editors. One of us (AC) screened all journal websites and documents sent by editors to determine whether editors specifically request these tasks. If the task requested by editors was not clear, we considered that editors did not request this task. For example, if editors requested “to evaluate the quality of the study,” we considered this task vague or unclear. However, if editors clearly requested the assessment of the randomization procedure or the strategy used to account for missing data or a blinding procedure, we considered the editor’s request clear, in this case, requesting the evaluation of risk of bias.

We also systematically recorded whether the guidelines were easily accessible (i.e., information accessible on the main page of the website), if the journal’s website had a specific peer review section, and if the website included a description of the peer review process.

### Statistical analysis

Quantitative data are described with means and SDs and categorical data with frequencies and percentages. Participant characteristics and classifications of tasks expected of peer reviewers are described overall and by participant competence (i.e., clinician, methodologist, or both). The classification was established by the mean ranking and proportion of participants who ranked the tasks in the first tertile in importance (i.e., ≥2 on the −5 to + 5 scale). We also performed a sensitivity analysis excluding the sub-group of peer reviewers who were first contacted because they were editors. These participants were first asked to provide details of their journal recommendations to reviewers and then asked again to rank the order of importance of the different tasks (Additional file 4). Statistical analysis involved use of SAS 9.3 (SAS Inst. Inc., Cary, NC).

## Results

### Identification of tasks expected from peer reviewers

We retrieved 2 117 citations from our search of articles related to tasks for peer reviewers; 98 full-text articles were selected and 90 were evaluated. Overall, among the 90 articles selected and evaluated, 48 were secondarily excluded because they did not report any recommendation or tasks dedicated to peer reviewers, 34 reported specific recommendations and tasks, and 8 referred to other sources (such as online training resources, website, etc.) from which further data were extracted (Fig. 1). The selected articles are in Additional file 5.

Overall, we selected 171 journals. All journals had a website. In total, 95 (56 %) journals did not have recommendations for peer reviewers available on their website and publisher interface; 34 (20 %) had recommendations only on their website, 32 (18 %) only on the publisher interface, and 10 (6 %) on both. Only 52 journals (30 %) had a specific peer reviewer section. Of the 171 journal editors contacted, 77 (45 %) responded and 69 (40 %) agreed to send us the documents they send to reviewers (Fig. 1), but three refused because they considered this information confidential. Among those who sent us documents, 44 (57 %) provided specific documents to their peer reviewers that are not available on their website. Among these specific documents, only 22 (50 %) gave more information than the website.

From all sources evaluated and after deletion of duplicates, we recorded 205 tasks (Fig. 1; Additional file 2). These tasks were combined into 36 principal tasks. These items concerned the following domains: etiquette/objectives (n = 3), abstract (n = 2), rationale (n = 2), methods (n = 6), statistics (n = 2), results (n = 4), discussion (n = 4), conclusions (n = 1), figures/tables (n = 2), references (n = 1), reporting guidelines (n = 2), trial registration (n = 2), presentation (n = 2), ethics (n = 1), and fraud (n = 2) (Table 1).

**Table 1:** Thirty-six tasks created for the Q-sort survey

Etiquette objectives	To read the journals’ recommendations to reviewers
	To provide recommendations on publication (e.g., reject/revise/publish)
	To evaluate all appendices when available
Rationale	To evaluate the novelty of the study (i.e., does the trial add enough to what is already in the published literature)
	To evaluate the importance of the study (i.e., usefulness for clinical practice)
Methods	To evaluate if the control group is appropriate
	To evaluate the risk of bias of the trial
	To evaluate the adequacy of the selection of participants and clinical setting
	To check if the intervention is described with enough details to allow replication
	To evaluate the relevance of the primary outcome(s)
	To evaluate the reliability and validity of the outcome measures
Trial registration	To compare information recorded on a clinical trials register such as ClinicalTrials.gov and reported in the manuscript
	To compare information recorded in the trial protocol when provided by the authors and reported in the manuscript
Reporting guidelines	To check if the items requested by the CONSORT statement are adequately reported by authors
	To check if items requested by the CONSORT extensions (e.g., cluster, non-pharmacologic treatments etc.) are adequately reported when appropriate
Ethic	To check if the study reported ethics review board approval
Statistics	To evaluate the adequacy of statistical analyses
	To check the sample size calculation
Results	To search for any inconsistencies or errors in the manuscript
	To search for any attempt to distort the presentation or interpretation of results (e.g., data “beautification”, spin, selective reporting)
	To check if all outcomes are adequately reported (results for each group, and the estimated effect size and its precision such as 95 % confidence interval)
	To check if all adverse events are adequately reported (participant withdrawals due to harms, absolute risk per arm and per adverse event type, grade, and seriousness)
Discussion	To evaluate if the discussion is consistent with the results
	To check if the authors referenced all important studies
	To check that limitations are adequately reported
	To discuss the results in relation to other studies
Conclusion	To determine whether the manuscript conclusion is consistent with the results
Fraud	To search for plagiarism or imitation in the paper
	To evaluate if the manuscript can be suspected of fraud
Figures tables	To check if all figures and tables are consistent with the text
	To evaluate whether figures and tables can be understood without having to refer the text
References	To evaluate if authors respect the requested format for references
Presentation	To evaluate the adequacy of the language (grammar, style, misspelling)
	To evaluate clarity of presentation
Abstract	To check if the authors reported all important outcomes and adverse events in the abstract
	To evaluate if the abstract conclusion is consistent with the results

### Survey of peer reviewers to sort the statements related to tasks by importance

#### Participants

We identified 7 996 email addresses useful for inviting participants to sort tasks; 229 participants completed the Q-sort survey of which 26 were excluded because they had not peer-reviewed at least one RCT report. Therefore, 203 were included in the final analysis. The responder proportions were as follows: 21 (10.3 %) from the editors panel, 40 (19.7 %) from the reviewers panel, 142 (70 %) from corresponding authors, 57 (40 %) being corresponding authors of *Public Environmental and Occupational Health* and other biostatistical journals, and 85 corresponding authors of RCTs indexed in PubMed.

Participant characteristics are in Table 2. Participants were mainly located in Europe (49 %) and USA/Canada (32.5 %) but also in South America (9.6 %) and Oceania (8.9 %). In total, 93 (45.8 %) had clinical expertise, 72 (35.5 %) were methodologists or statisticians, 17 (8.4 %) had both clinical and methodological expertise, and 21 (10.3 %) had expertise in another area (i.e., were researchers, engineers, economists). Most participants (92 %) were investigators of at least one RCT. Overall, 150 participants (73.9 %) reviewed one to five RCT reports per year. One third did not receive any form of training to perform a peer review. More than half of the participants (61.1 %, n = 124) spent more than 2 h peer-reviewing a manuscript. Half regularly agreed to disclose their name when proposed or requested by editors.

**Table 2:** Participant characteristics

Characteristic	Total
	N = 203
Expertise	N (%)
− Clinician	93 (45.8)
− Methodologist	72 (35.5)
− Both (clinician/methodologist)	17 (8.4)
− Other	21 (10.3)
Affiliation^a^	
− Non-profit	179 (95.7)
− For-profit	5 (2.7)
− Publisher	3 (1.6)
Country^b^	
− Oceania	14 (8.9)
− South America/Asia	15 (9.6)
− USA/Canada	51 (32.5)
− Europe	77 (49.0)
Total no. of completed randomized trials participated in as an investigator^c^	
−0	17 (8.4)
−1–5	99 (48.8)
−6–10	42 (20.7)
−11–15	19 (9.4)
−16–20	4 (2.0)
− >20	22 (10.8)
Mean no. of articles peer-reviewed per year^c^	
−1–5	35 (17.2)
−6–10	51 (25.1)
−11–20	55 (27.1)
−20–50	39 (19.2)
− >50	23 (11.3)
Mean no. of articles reporting an RCT peer-reviewed per year^c^	
−1–5	150 (73.9)
−6–10	27 (13.3)
−11–20	13 (6.4)
−20–50	8 (3.9)
− >50	5 (2.5)
Ask colleagues to help with the peer review^c^	
− Never	58 (28.6)
− Rarely	92 (45.3)
− Sometimes	41 (20.2)
− Regularly	10 (4.9)
− Always	2 (1.0)
Training^d^	
− Formal academic training	33 (16.4)
− Mentoring by your supervisor	59 (29.4)
− Tutorial on training sessions offered by editors	13 (6.5)
− Not trained	61 (30.3)
− Other trained	2 (1.0)
− Several forms of training	33 (16.4)
Mean time for a peer review of an RCT (hours)^c^	
− <1	7 (3.4)
−1–2	72 (35.5)
−2–4	85 (41.9)
− >4	39 (19.2)
Agree to disclose your name when proposed or requested by an editor^c^	
− Never	25 (12.3)
− Rarely	25 (12.3)
− Sometimes	49 (24.1)
− Regularly	45 (22.2)
− Always	59 (29.1)

^a^12 (5.9 %) missing data; ^b^46 (22.7 %) missing data; ^c^No missing data; ^d^2 (1 %) missing data

*RCT* Randomized controlled trial

### Sorting tasks

Table 3 reports the tasks sorted by participants. The nine tasks rated as the most important were to 1) evaluate the risk of bias of the trial, 2) determine whether the manuscript conclusion is consistent with the results, 3) evaluate the adequacy of the statistical analyses, 4) evaluate whether the control group is appropriate, 5) check if all outcomes are adequately reported, 6) evaluate the relevance of the primary outcome(s), 7) search for any attempt to distort the presentation or interpretation of results, 8) evaluate the reliability and validity of the outcome measures, and 9) evaluate the importance of the study. For clinicians, the 2nd and 3rd most important tasks were to evaluate the importance of the study and the reliability and validity of the outcome measures, whereas for methodologists, these tasks were ranked lower, 14th and 11th, respectively. The task to evaluate the adequacy of statistical analyses was ranked 2nd by methodologists but 6th by clinicians. The sensitivity analysis excluding the answers of the 21 participants invited because they were editors showed consistent results (see Additional file 4).

**Table 3:** Tasks sorted by participants in the Q-sort survey. Data are (overall rank) and mean ± SD rank of participants

	Total	Clinician	Methodologist	Both	Other
	(N = 203)	(N = 93)	(N = 72)	(N = 17)	(N = 21)
To evaluate the risk of bias of the trial	**(1)** 2.1 ± 2.0	**(5)** 1.3 ± 2.1	**(1)** 2.8 ± 1.8	**(1)** 3.6 ± 0.8	**(1)** 2.5 ± 1.6
To determine if the manuscript conclusion is consistent with the results	**(2)** 1.9 ± 1.9	**(1)** 1.7 ± 2.0	**(3)** 2.1 ± 1.8	**(2)** 2.4 ± 2.1	**(3)** 1.9 ± 1.9
To evaluate the adequacy of statistical analyses	**(3)** 1.8 ± 1.7	**(6)** 1.2 ± 1.6	**(2)** 2.6 ± 1.7	**(3)** 2.2 ± 1.6	**(5)** 1.6 ± 1.5
To evaluate if the control group is appropriate	**(4)** 1.4 ± 1.8	**(8)** 1.1 ± 1.7	**(4)** 1.9 ± 1.8	**(8)** 1.2 ± 1.4	**(6)** 1.4 ± 2.1
To check if all outcomes are adequately reported	**(5)** 1.4 ± 2.0	**(9)** 1.1 ± 2.1	**(5)** 1.7 ± 1.9	**(5)** 1.5 ± 1.6	**(4)** 1.9 ± 1.9
To evaluate the relevance of the primary outcome(s)	**(6)** 1.3 ± 2.1	**(4)** 1.3 ± 2.1	**(6)** 1.5 ± 2.1	**(14)** 0.6 ± 2.6	**(8)** 1.1 ± 1.7
To search for any attempt to distort the presentation or interpretation of results	**(7)** 1.2 ± 2.0	**(11)** 0.9 ± 2.0	**(7)** 1.3 ± 1.8	**(4)** 1.9 ± 2.5	**(2)** 2.2 ± 2.1
To evaluate the reliability and validity of the outcome measures	**(8)** 1.1 ± 2.0	**(3)** 1.4 ± 2.2	**(11)** 0.9 ± 1.9	**(12)** 0.7 ± 2.1	**(9)** 1.0 ± 1.9
To evaluate the importance of the study	**(9)** 1.1 ± 2.4	**(2)** 1.7 ± 2.4	**(14)** 0.6 ± 2.5	**(17)** -0.1 ± 2.0	**(13)** 0.7 ± 2.0
To evaluate if the abstract conclusion is consistent with the results	**(10)** 1.0 ± 1.9	**(10)** 1.0 ± 2.1	**(9)** 1.2 ± 1.7	**(9)** 1.2 ± 1.9	**(20)** 0.0 ± 1.7
To evaluate if the discussion is consistent with the results	**(11)** 1.0 ± 1.8	**(7)** 1.2 ± 1.7	**(13)** 0.7 ± 1.7	**(13)** 0.7 ± 1.9	**(7)** 1.2 ± 2.1
To check if all adverse events are adequately reported	**(12)** 0.9 ± 1.9	**(14)** 0.7 ± 2.0	**(8)** 1.2 ± 1.7	**(6)** 1.4 ± 1.9	**(10)** 0.9 ± 2.1
To check if the intervention is described with enough details to allow replication	**(13)** 0.8 ± 1.8	**(16)** 0.6 ± 1.8	**(10)** 1.1 ± 1.7	**(10)** 1.0 ± 1.8	**(18)** 0.2 ± 1.9
To check that limitations are adequately reported	**(14)** 0.7 ± 1.7	**(17)** 0.4 ± 1.8	**(12)** 0.8 ± 1.6	**(7)** 1.3 ± 1.7	**(11)** 0.9 ± 1.6
To evaluate the adequacy of the selection of participants and clinical setting	**(15)** 0.5 ± 1.9	**(13)** 0.7 ± 1.9	**(15)** 0.5 ± 1.8	**(15)** 0.4 ± 1.7	**(17)** 0.2 ± 2.0
To search for any inconsistencies or errors in the manuscript	**(16)** 0.3 ± 2.2	**(21)** 0.0 ± 2.1	**(17)** 0.3 ± 2.2	**(11)** 1.0 ± 2.4	**(14)** 0.6 ± 2.0
To evaluate the novelty of the study	**(17)** 0.2 ± 2.4	**(12)** 0.8 ± 2.5	**(20)** –0.1 ± 2.0	**(21)** –0.3 ± 2.1	**(24)** –0.5 ± 2.7
To check the sample size calculation	**(18)** 0.2 ± 2.1	**(18)** 0.4 ± 1.9	**(18)** –0.1 ± 2.4	**(24)** –0.5 ± 2.0	**(15)** 0.5 ± 2.1
To check if the authors reported all important outcomes and adverse events in the abstract	**(19)** 0.1 ± 2.1	**(22)** 0.0 ± 2.3	**(16)** 0.4 ± 1.9	**(20)** –0.2 ± 1.8	**(23)** –0.3 ± 2.3
To discuss the results in relation to other studies	**(20)** 0.0 ± 1.9	**(19)** 0.3 ± 2.0	**(19)** –0.1 ± 1.6	**(30)** –1.0 ± 2.1	**(19)** 0.1 ± 2.0
To evaluate if the manuscript can be suspected of fraud	**(21)** –0.1 ± 2.6	**(15)** 0.7 ± 2.7	**(30)** –1.1 ± 2.4	**(29)** –0.9 ± 1.6	**(12)** 0.9 ± 2.3
To provide recommendations on publication	**(22)** –0.2 ± 2.5	**(20)** 0.3 ± 2.8	**(23)** –0.5 ± 2.2	**(22)** –0.4 ± 2.3	**(26)** –0.7 ± 2.3
To check if all figures and tables are consistent with the text	**(23)** –0.2 ± 1.7	**(23)** –0.1 ± 1.7	**(21)** –0.4 ± 1.6	**(19)** –0.2 ± 2.0	**(21)** –0.1 ± 1.6
To evaluate clarity of presentation	**(24)** –0.5 ± 1.9	**(26)** –0.5 ± 2.0	**(22)** –0.5 ± 1.9	**(28)** –0.8 ± 1.6	**(27)** –0.7 ± 1.7
To check if the study reported ethics review board approval	**(25)** –0.5 ± 2.2	**(25)** –0.3 ± 2.3	**(27)** –0.9 ± 2.1	**(23)** –0.4 ± 2.1	**(22)** –0.3 ± 2.3
To search for plagiarism or imitation in the paper	**(26)** –0.7 ± 2.3	**(24)** –0.1 ± 2.4	**(33)** –1.4 ± 2.2	**(32)** –1.4 ± 2.0	**(16)** 0.2 ± 1.9
To compare information recorded in the trial protocol when provided by the authors and reported in the manuscript	**(27)** –0.7 ± 2.3	**(27)** –0.8 ± 2.2	**(24)** –0.6 ± 2.5	**(18)** –0.2 ± 2.2	**(30)** –1.0 ± 2.1
To check if the items requested by the CONSORT Statement are adequately reported by authors	**(28)** –0.9 ± 2.3	**(29)** –1.1 ± 2.2	**(26)** –0.8 ± 2.2	**(16)** 0.1 ± 2.6	**(25)** –0.5 ± 2.6
To check if the authors referenced all important studies	**(29)** –1.1 ± 1.7	**(28)** –1.1 ± 1.8	**(25)** –0.8 ± 1.7	**(33)** –1.6 ± 1.6	**(33)** –1.5 ± 1.2
To evaluate whether figures and tables can be understood without having to refer the text	**(30)** –1.1 ± 1.9	**(30)** –1.1 ± 1.9	**(31)** –1.2 ± 1.8	**(26)** –0.7 ± 2.0	**(29)** –1.0 ± 2.2
To check if items requested by the CONSORT extensions are adequately reported when appropriate	**(31)** –1.2 ± 1.9	**(32)** –1.5 ± 2.0	**(29)** –1.1 ± 1.8	**(25)** –0.7 ± 1.6	**(28)** –0.8 ± 2.0
To compare information recorded on a clinical trials register such as ClinicalTrials.gov and reported in the manuscript	**(32)** –1.3 ± 2.1	**(31)** –1.5 ± 2.1	**(28)** –1.0 ± 2.3	**(31)** –1.4 ± 1.7	**(32)** –1.5 ± 2.3
To read the journals’ recommendations to reviewers	**(33)** –1.4 ± 2.0	**(33)** –1.7 ± 1.9	**(32)** –1.2 ± 1.7	**(27)** –0.8 ± 2.5	**(31)** –1.1 ± 2.6
To evaluate all appendices when available	**(34)** –2.3 ± 1.6	**(34)** –2.2 ± 1.8	**(34)** –2.5 ± 1.4	**(34)** –1.8 ± 1.8	**(34)** –2.8 ± 1.1
To evaluate the adequacy of the language	**(35)** –2.9 ± 1.7	**(35)** –2.7 ± 1.9	**(35)** –3.0 ± 1.5	**(35)** –3.1 ± 1.3	**(35)** –3.4 ± 1.4
To evaluate if authors respect the requested format for references	**(36)** –4.0 ± 1.4	**(36)** –3.8 ± 1.6	**(36)** –4.0 ± 1.3	**(36)** –4.4 ± 1.2	**(36)** –4.0 ± 1.4

### Assessment of tasks requested in editors’ recommendations to peer reviewers

**Fig. 2:**
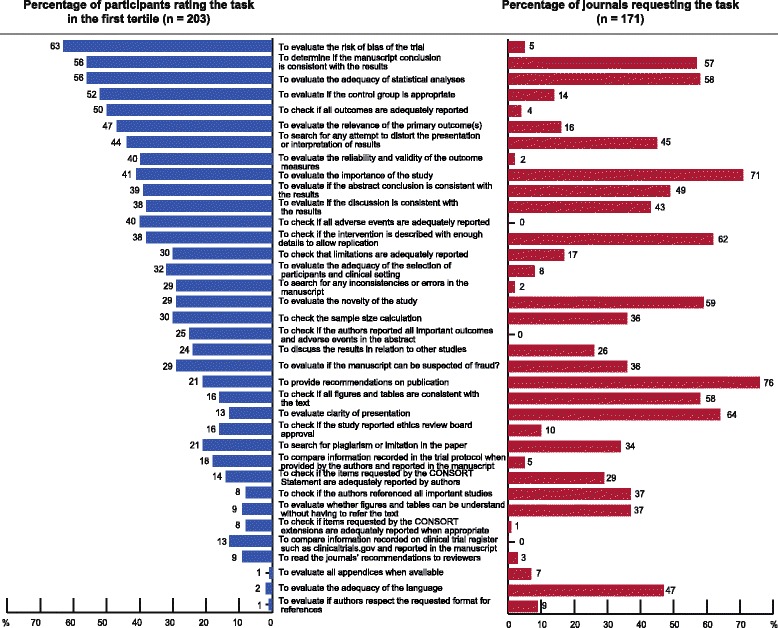
Representation for each task of the proportion of participants rating the task in the first tertile (i.e., ≥2 on the scale from −5 to +5 for each task) and the proportion of editors requesting the task in their recommendations to authors. The tasks are sorted according to the mean ranking of participants

The tasks rated in the first tertile of importance (≥2 on the −5 to +5 scale) were not completely congruent with the tasks most frequently requested by editors (Fig. 2). For example, evaluating the risk of bias was rated in the first tertile by 63 % of participants but clearly requested by only 5 % of editors. In contrast, the task most frequently requested by editors was the recommendation for publication (76 %), but this task was classified in the first tertile by only 21 % of all participants.

Among the 171 websites studied, only 76 (44 %) had recommendations for peer reviewers. These recommendations were easily accessible for only half of the websites (n = 38). One third had had a specific peer review section. However, 70 % (124) included a description of the peer review process.

## Discussion

This study involved the identification and sorting of all tasks expected from peer reviewers when evaluating the report of an RCT. We performed a large systematic review of the literature and editors’ guidelines and surveyed peer reviewers from various backgrounds who evaluated peer reviewer tasks by the Q-sort method.

Overall, we identified 221 different tasks that could be expected from peer reviewers when evaluating an RCT report. These tasks involved different levels of expertise. For example, assessing the adequacy of statistical analysis involves methodological and statistical expertise, whereas assessing the adequacy of the selection of participants and clinical setting involves content expertise. The tasks rated as most important were related to the methodology (evaluating the risk of bias of the trial, whether the control group is appropriate, and the reliability and validity of the outcome measures), statistics (evaluating the adequacy of statistical analyses), and results (determining whether the manuscript conclusions are consistent with the results and whether all outcomes are adequately reported, and searching for any attempt to distort the presentation or interpretation of results). We found some differences in how tasks were sorted according to the peer reviewers’ expertise (clinicians, methodologists, or both). Therefore, peer reviewers will place different values on different tasks according to their expertise.

We found some differences between tasks clearly requested by editors in their recommendations and tasks rated as important by reviewers. Editors frequently ask whether an article is suitable for publication, but reviewers ranked this task 22nd among the 36 tasks. Editors had more recommendations about the format (tables/figures/presentation) and the importance of the study than the methodology. In contrast, the tasks considered the least important by peer reviewers mainly related to the format (evaluating whether authors respect the requested format for references and the adequacy of the language).

Our findings also reflect that current recommendations by editors may not be sufficiently explicit. Editors’ recommendations were mainly generic and not for a specific type of study design. This is probably related to the fact that editors usually rely on the expertise of reviewers and expect that the reviewers know which aspects are important and which aspects need to be evaluated. However, some evidence in the literature shows that the impact of peer reviewers is questionable [9, 24–29]. This situation is problematic because peer reviewers are usually not specifically trained; they have limited time for peer review and receive minimal rewards for this task. Further, they may misunderstand what they are expected to do. Moreover, there may be ambiguities regarding who should perform a specific task. Some tasks, such as checking the quality of reporting or checking trial registration, can be considered by editors as a reviewer task and by reviewers as an editorial task. Editors could try to achieve consensus on what they expect from peer reviewers when evaluating the report of an RCT.

Furthermore, important and simple tasks that could easily increase a paper’s quality were poorly rated by peer reviewers and rarely requested by editors. In fact, the task dedicated to trial registration (i.e., the item to compare information recorded on a clinical trial register such as ClinicalTrials.gov and reported in the manuscript) was classified 32nd among the 36 condensed items and never clearly requested by editors. This situation raises some concern because trial registration may limit the risk of selective reporting bias [30]. However, these results are consistent with other studies. In fact, Mathieu et al. [22] showed that only one third of peer reviewers examined registered trial information and reported any discrepancies to journal editors. These results could be linked to the ambiguity on who, editors or reviewers, is responsible for this task. The medical community should be aware of the importance of trial registration and ensure that proper information is submitted to registries. There may be legitimate discrepancies between the manuscript and registry record, but these should be transparently reported in the paper or in the registry.

Similarly, the tasks dedicated to reporting guidelines (i.e., checking whether the items requested by the CONSORT Statement are adequately reported by authors and whether items requested by the CONSORT Statement extensions are adequately reported when appropriate) were classified 28th and 31st, respectively, among the 36 items and requested by 29 % and 1 % of editors, respectively. Some studies have demonstrated the positive impact of reporting guidelines, such as the CONSORT guidelines, on reporting quality [31]. The ranking of tasks concerning the CONSORT Statement could explain in part why reporting according to the CONSORT guidelines is poor [7, 32].

Our study has some limitations. First, we cannot know whether peer reviewers who participated in the study are representative of all peer reviewers. In fact, we cannot estimate the response rate because the emails sent may have been identified as spam or sent to junk mail folders. However, this survey focuses on qualitative input and it was mainly important to have a variety of expertise. Second, our sample included only 171 journals, with a response rate of journal editors of 45 %. Third, only one author extracted data on the tasks expected of peer reviewers (AC). However, two reviewers achieved consensus on the classification of these tasks. Fourth, our study was a snapshot of editors’ recommendations, and some journals may have since updated their websites and their instructions to peer reviewers (30 July, 2014). Finally, although we performed a systematic methodological review, we cannot exclude that we missed some reports on this topic.

## Conclusions

The tasks considered most important to peer reviewers (i.e., the methodology, statistics, and results) were frequently not clearly requested by journal editors.

## Additional files

Additional file 1: 1.1. Journals publishing at least 15 randomized controlled trial (RCT) reports (search date February 2, 2014) indexed in PubMed with the keywords *randomized controlled trial* with the following filters: study type RCT, English language, human studies, published between January 1 and March 31, 2013, and with an abstract available. **1.2.** The 10 journals with the highest impact factor (Journal Citation Report 2012) for 14 medical areas. Only journals publishing at least one RCT report in the three last issues were included. **1.3.** Duplicate journals. Additional file 2:
**The 205 tasks after removing duplicates.**
Additional file 3:
**Example of sorting with Q-sort.**
Additional file 4:
**Sensitivity analysis of task ranking with and without the editor panel.**
Additional file 5:
**Articles selected during the literature review.**
