# Supplementary material for: The most important tasks for peer reviewers evaluating a randomized controlled trial are not congruent with the tasks most often requested by journal editors
Source: BMC Med. 2015 Jul 3;13:158. doi: 10.1186/s12916-015-0395-3 (PMC4491236; doi:10.1186/s12916-015-0395-3)
Supplement: Additional file 1: 1.1. — Journals publishing at least 15 randomized controlled trial (RCT) reports (search date February 2, 2014) indexed in PubMed with the keywords randomized controlled trial with the following filters: study type RCT, English language, human studies, published between January 1 and March 31, 2013, and with an abstract available. 1.2. The 10 journals with the highest impact factor (Journal Citation Report 2012) for 14 medical areas. Only journals publishing at least one RCT report in the three last issues were included. 1.3. Duplicate journals. [file 12916_2015_395_MOESM1_ESM.doc]

Additional file 1 **1.1.** Journals publishing at least 15 randomized controlled trial (RCT) reports (search date February 2, 2014) indexed in PubMed with the keywords *randomized controlled trial* with the following filters: study type RCT, English language, human studies, published between January 1, 2013 and March 31, 2013, and with an abstract available

| **Journal's Name** | **Impact Factor** |
| --- | --- |
| Accident Analysis and Prevention | 1,964 |
| Acta Anaesthesiologica Scandinavica | 2,355 |
| Addictive Behaviors | 2,021 |
| Addiction | 4,746 |
| AIDS and Behavior | 2,979 |
| Alcoholism- Clinical and Experimental Research | 3,421 |
| American Heart Journal | 4,497 |
| American Journal of Cardiology | 3,209 |
| American Journal of Clinical Nutrition | 6,504 |
| American Journal of Kidney Diseases | 5,294 |
| American Journal of Ophtalmology | 3,631 |
| Anesthesia and Analgesia | 3,300 |
| Annals of Oncology | 7,384 |
| Antimicrobial Agents and Chemotherapy | 4,565 |
| Appetite | 2,541 |
| Archives of Gynecology and Obstetrics | 1,330 |
| Archives of Physical Medicine and Rehabilitation | 2,358 |
| Atherosclerosis | 3,991 |
| Behaviour Research and Therapy | 3,471 |
| BJOG- an International Journal Of Obstetrics and gynaecology | 3,760 |
| BJU International | 3,046 |
| Blood | 9,060 |
| BMC Musculoskeletal Disorders | 1,875 |
| BMC Psychiatry | 2,233 |
| BMC Public Health | 2,076 |
| British Journal of Anaesthesia | 4,237 |
| British Journal of Cancer | 5,082 |
| British Journal of Clinical Pharmacology | 3,578 |
| British Journal of Nutrition | 3,302 |
| Cancer | 4,901 |
| Circulation- Heart Failure | 6,684 |
| Circulation | 15,202 |
| Clinical Infectious Diseases | 9,374 |
| Clinical Interventions in Aging | 2,651 |
| Clinical Journal of the American Society of Nephrology | 5,068 |
| Clinical Journal of Pain | 2,552 |
| Clinical Oral Investigations | 2,200 |
| Clinical Rehabilitation | 2,191 |
| Clinical Therapeutics | 2,230 |
| Contemporary Clinical Trials | 1,597 |
| Diabetes Care | 7,735 |
| Diabetes Obesity & Metabolism | 5,181 |
| Drug and Alcohol Dependence | 3,141 |
| European Heart Journal | 14,097 |
| European Journal of Applied Physiology | 2,660 |
| European Journal of Cancer | 5,061 |
| European Journal of Clinical Nutrition | 2,756 |
| European Journal of Clinical Pharmacology | 2,741 |
| European Journal of Heart Failure | 5,247 |
| Experimental Brain Research | 2,221 |
| Journal of Affective Disorders | 3,295 |
| Journal of Alternative and Complementary Medicine | 1,464 |
| Journal of the American College of Cardiology | 14,086 |
| Journal of Applied Physiology | 2,660 |
| Journal of Clinical Endocrinology and Metabolism | 6,430 |
| Journal of Clinical Oncology | 18,038 |
| Journal of Clinical Periodontology | 3,688 |
| Journal of Clinical Pharmacology | 2,963 |
| Journal of Dentistry | 3,200 |
| Journal of Drugs in Dermatology | 1,161 |
| Journal of Infectious Diseases | 5,848 |
| Journal of Medical Internet Research | 3,768 |
| Journal of Nutrition | 4,196 |
| Journal of Pediatric | 4,035 |
| Journal of Sports Science | 2,082 |
| Journal of Strength and Conditioning Research | 1,795 |
| Journal of Substance Abuse Treatment | 1,985 |
| Journal of Urology | 3,696 |
| JAMA | 29,978 |
| Lancet | 39,060 |
| Lancet Oncology | 25,117 |
| Metabolism | 4,514 |
| New England Journal of Medicine | 51,658 |
| Neurorehabilitation and Neural Repair | 4,278 |
| Nicotine & Tobacco Research | 2,477 |
| Nutrition | 2,859 |
| Obesity (Silver Spring) | 3,491 |
| Osteoporosis International | 4,039 |
| Pain | 5,644 |
| Pediatrics | 5,119 |
| PLoS One | 3,730 |
| Psychology & Health | 1,950 |
| Psychopharmacology (Berl) | 4,595 |
| Respiratory Medicine | 2,585 |
| Schizophrenia Research | 4,590 |
| Stroke | 6,158 |
| Surgical Endoscopy and Other Interventional Techniques | 3,427 |
| Trials | 2,206 |
| Vaccine | 3,492 |
| Zh Nevrol Psikhiatr Im S S Korsakova | NA |
| Zhongguo Zhen Jiu | NA |

**1.2.** The 10 journals with the highest impact factor (Journal Citation Report 2012) for 14 medical areas. Only journals publishing at least one RCT report in the 3 last issues were included.

| **Journal's name** | **Impact Factor** | **Specialties** | **Selected if at least 3 RCTs of clinical trials in 3 last issues** |
| --- | --- | --- | --- |
| Anaesthesia | 3,486 | Anesthesiology | Yes |
| Anesthesia and Analgesia | 3,300 | Anesthesiology | Yes |
| Anesthesiology | 5,163 | Anesthesiology | Yes |
| British Journal of Anaesthesia | 4,237 | Anesthesiology | Yes |
| European Journal of Anaesthesiology | 2,792 | Anesthesiology | Yes |
| European Journal of Pain | 3,067 | Anesthesiology | Yes |
| Minerva Anestesiologica | 2,818 | Anesthesiology | Yes |
| Pain | 5,644 | Anesthesiology | Yes |
| Pain Practice | 2,605 | Anesthesiology | Yes |
| Regional Anesthesia and Pain Medicine | 3,464 | Anesthesiology | Yes |
| Circulation | 15,202 | Cardiac & Cardiovascular systems | Yes |
| Circulation- Cardiovascular Genetics | 6,728 | Cardiac & Cardiovascular systems | Yes |
| Circulation- Cardiovascular Interventions | 6,543 | Cardiac & Cardiovascular systems | Yes |
| Circulation- Heart Failure | 6,684 | Cardiac & Cardiovascular systems | Yes |
| Circulation Research | 11,861 | Cardiac & Cardiovascular systems | Yes |
| European Heart Journal | 14,097 | Cardiac & Cardiovascular systems | Yes |
| JACC- Cardiovascular Imaging | 6,164 | Cardiac & Cardiovascular systems | Yes |
| JACC- Cardiovascular Interventions | 6,543 | Cardiac & Cardiovascular systems | Yes |
| Journal of the American College of Cardiology | 14,086 | Cardiac & Cardiovascular systems | Yes |
| Nature Reviews in Cardiology | 10,400 | Cardiac & Cardiovascular systems | No |
| Acta Neuropathologica | 9,734 | Clinical neurology | No |
| Alzheimers & Dementia | 14,483 | Clinical neurology | Yes |
| Annals of Neurology | 11,193 | Clinical neurology | Yes |
| Archives of Neurology | 7,685 | Clinical neurology | No |
| Brain | 9,915 | Clinical neurology | Yes |
| Lancet Neurology | 23,917 | Clinical neurology | Yes |
| Nature Reviews Neurology | 15,518 | Clinical neurology | No |
| Neurology | 8,249 | Clinical neurology | Yes |
| Neuro-oncology | 6,180 | Clinical neurology | Yes |
| Sleep Medicine Reviews | 8,681 | Clinical neurology | Yes |
| American Journal of Respiratory and Critical Care Medicine | 11,041 | Critical Care Medecine | Yes |
| Chest | 5,854 | Critical Care Medecine | Yes |
| Critical Care | 4,718 | Critical Care Medecine | Yes |
| Critical Care Medicine | 6,124 | Critical Care Medecine | Yes |
| Current Opinion in Critical Care | 2,967 | Critical Care Medecine | No |
| Intensive Care Medicine | 5,258 | Critical Care Medecine | Yes |
| Journal of Neurotrauma | 4,295 | Critical Care Medecine | Yes |
| Minerva Anestesiologica | 2,818 | Critical Care Medecine | Yes |
| Neurocritical Care | 3,038 | Critical Care Medecine | Yes |
| Resucitation | 4,104 | Critical Care Medecine | Yes |
| Academic Emergency Medicine | 1,757 | Emergency Medicine | Yes |
| American journal of Emergency Medecine | 1,704 | Emergency Medicine | Yes |
| Annals of Emergency Medicine | 4,285 | Emergency Medicine | Yes |
| Emergencias | 2,578 | Emergency Medicine | Yes |
| Emergency Medicine Journal | 1,645 | Emergency Medicine | Yes |
| Injury | 2,174 | Emergency Medicine | Yes |
| Journal of Trauma - Injury infection and Critical Care | 2,348 | Emergency Medicine | No |
| Prehopospital Emergency Care | 1,859 | Emergency Medicine | Yes |
| Resuscitation | 4,104 | Emergency Medicine | Yes |
| Scandinavian Journal of Trauma Resuscitation & Medicine | 1,680 | Emergency Medicine | Yes |
| Arteriosclerosis Thrombosis and Vascular Biology | 6,338 | Hematology | Yes |
| Blood | 9,060 | Hematology | Yes |
| Blood Reviews | 6,000 | Hematology | Yes |
| Circulation Research | 11,861 | Hematology | Yes |
| Haematologica - The Hematology Journal | 5,935 | Hematology | No |
| Journal of Cerebral Blood Flow and Metabolism | 5,398 | Hematology | No |
| Journal of Thrombosis and Haemostasis | 6,081 | Hematology | No |
| Leukemia | 10,164 | Hematology | Yes |
| Stem Cells | 7,701 | Hematology | No |
| Thrombosis and Haemostasis | 6,094 | Hematology | Yes |
| AIDS | 6,407 | Infectious Diseases | Yes |
| Clinical Infectious Diseases | 9,374 | Infectious Diseases | Yes |
| Current Opinion in HIV and AIDS | 4,704 | Infectious Diseases | No |
| Current Opinion in Infectious Diseases | 4,870 | Infectious Diseases | No |
| Emerging Infectious Diseases | 5,993 | Infectious Diseases | Yes |
| Eurosurveillance | 5,491 | Infectious Diseases | Yes |
| JAIDS- Journal of Acquired immune Deficinecy Syndromes | 4,653 | Infectious Diseases | Yes |
| Journal of Antimicrobial Chemotherapy | 5,338 | Infectious Diseases | Yes |
| Journal of Infectious Diseases | 5,848 | Infectious Diseases | Yes |
| Lancet Infectious Diseases | 19,966 | Infectious Diseases | Yes |
| Annals of Internal Medicine | 13,976 | Medicine, General & Internal | Yes |
| Archives of Internal Medicine | 10,579 | Medicine, General & Internal | Yes |
| BMC Medicine | 6,679 | Medicine, General & Internal | Yes |
| British Medical Journal | 17,215 | Medicine, General & Internal | Yes |
| Canadian Medical Association Journal | 6,465 | Medicine, General & Internal | Yes |
| JAMA | 29,978 | Medicine, General & Internal | Yes |
| Journal of Internal Medicine | 6,455 | Medicine, General & Internal | Yes |
| Lancet | 39,060 | Medicine, General & Internal | Yes |
| New England Journal of Medicine | 51,658 | Medicine, General & Internal | Yes |
| Plos Medicine | 15,253 | Medicine, General & Internal | Yes |
| Annals of Neurology | 11,193 | Neurosciences | Yes |
| Annual Review of Neuroscience | 20,614 | Neurosciences | No |
| Behavioral and Brain Sciences | 18,571 | Neurosciences | Yes |
| Brain | 9,915 | Neurosciences | Yes |
| Molecular Psychiatry | 14,897 | Neurosciences | Yes |
| Nature Neurosciences Neurosci | 15,251 | Neurosciences | No |
| Nature Reviews Neuroscience | 31,673 | Neurosciences | No |
| Neuron | 15,766 | Neurosciences | Yes |
| Trends in Cognitive sciences | 16,008 | Neurosciences | No |
| Trends Neurosci | 13,582 | Neurosciences | Yes |
| Biochimica et Biopsysica - Reviews of Cancer | 9,033 | Oncology | No |
| CA- A Cancer Journal for Clinicians | 153,459 | Oncology | Yes |
| Cancer Cell | 24,755 | Oncology | No |
| Cancer Discovery | 10,143 | Oncology | Yes |
| JNCI- Journal of the National Cancer Institute | 14,336 | Oncology | Yes |
| Journal of Clinical Oncology | 18,038 | Oncology | Yes |
| Lancet Oncology | 25,117 | Oncology | Yes |
| Leukemia | 10,174 | Oncology | Yes |
| Nature Reviews Cancer | 35,000 | Oncology | No |
| Nature Reviews Clinical Oncology | 14,771 | Oncology | No |
| Archives of Disease in Childhood | 3,051 | Pediatrics | Yes |
| Archives of Disease in Childhood- Fetal and Neonatal Edition | 3,451 | Pediatrics | Yes |
| Archives Pediatrics Adolescent Medicine | 4,282 | Pediatrics | Yes |
| European Child & Adolescent Psychiatry | 3,699 | Pediatrics | Yes |
| Journal of Pediatric | 4,035 | Pediatrics | Yes |
| Journal of the American Academy of Child and Adolescent Psychiatry | 6,970 | Pediatrics | Yes |
| Pediatric Allergy and Immunology | 3,376 | Pediatrics | Yes |
| Pediatrics | 5,119 | Pediatrics | Yes |
| Seminars in Fetal & Neonatal Medicine | 3,505 | Pediatrics | No |
| The Pediatric Infectious Disease Journal | 3,569 | Pediatrics | Yes |
| American journal of Psychaitry | 14,721 | Psychiatry | Yes |
| Archives of General Psychaitry | 13,772 | Psychiatry | No |
| Biological psychiatry | 9,247 | Psychiatry | No |
| British Journal of Psychiatry | 6,606 | Psychiatry | No |
| Journal of Psychiatry & Neuroscience | 6,242 | Psychiatry | No |
| Molecular Psychiatry | 14,897 | Psychiatry | Yes |
| Neuropsychopharmacology | 8,678 | Psychiatry | No |
| Psychoterapy and Psychosomatics | 7,230 | Psychiatry | No |
| Schizophrenia Bulletin | 8,486 | Psychiatry | No |
| World Psychiatry | 8,974 | Psychiatry | No |
| Annals of Rheumatic Diseases | 9,111 | Rheumatology | Yes |
| Arthritis and Rheumatology | 7,477 | Rheumatology | Yes |
| Arthritis Care & Research | 3,731 | Rheumatology | Yes |
| Arthritis Research & Therapy | 4,302 | Rheumatology | Yes |
| Best Practice & Research in Clinical Rheumatology | 3,550 | Rheumatology | Yes |
| Curr Opin Rheumatol | 5,191 | Rheumatology | No |
| Nature Reviews Rheumatology | 9,745 | Rheumatology | No |
| Osteoarthritis and Cartilage | 4,262 | Rheumatology | Yes |
| Rheumatology | 4,212 | Rheumatology | Yes |
| Seminars in Arthritis and Rheumatism | 3,806 | Rheumatology | Yes |
| American Journal of surgical pathology | 4,868 | Surgery | Yes |
| American Journal of Transplantation | 6,192 | Surgery | Yes |
| Annals of Surgery | 6,329 | Surgery | Yes |
| Annals Surgical Oncology | 4,120 | Surgery | Yes |
| Archives of Surgery |  | Surgery | Yes |
| British Journal of Surgery | 4,839 | Surgery | Yes |
| Endoscopy | 5,735 | Surgery | Yes |
| Journal of neurology Neurosurgery and Psychiatry | 4,924 | Surgery | Yes |
| Journal of the American College of Surgeons | 4,500 | Surgery | Yes |
| Surgery for obesity and Related Diseases | 4,121 | Surgery | Yes |

**1.3.** Duplicate journals

| Annals of Neurology |
| --- |
| Brain |
| Leukemia |
| Circulation Research |
| Minerva Anestesiol |
| Anesthesia and Analgesia |
| Atherosclerosis |
| Blood |
| British Journal of Anaesthesia |
| Circulation- Heart Failure |
| Circulation |
| Clinical Infectious Diseases |
| European Heart Journal |
| Journal of the American College of Cardiology |
| Journal of Clinical Oncology |
| Journal of Infectious Diseases |
| Journal of Pediatric |
| JAMA |
| Lancet |
| Lancet Oncology |
| New England Journal of Medicine |
| Pain |
| Pediatrics |
| Molecular Psychiatry |
