# Supplementary material for: The most important tasks for peer reviewers evaluating a randomized controlled trial are not congruent with the tasks most often requested by journal editors
Source: BMC Med. 2015 Jul 3;13:158. doi: 10.1186/s12916-015-0395-3 (PMC4491236; doi:10.1186/s12916-015-0395-3)
Supplement: Additional file 2: — The 205 tasks after removing duplicates. [file 12916_2015_395_MOESM2_ESM.doc]

Additional file 2. The 205 tasks after removing duplicates

| **Logistics** | 1 | Complete the review by the assigned deadline |
| --- | --- | --- |
|  | 2 | Organize notations by sections |
|  | 3 | When making critical notes on particular sections of a paper: indicate location of the section in question as specifically as possible |
|  | 4 | Provide separate comments to the editors and to the author |
| **Etiquette/Objectives** | 5 | Provide constructive and useful comments |
|  | 6 | Supply evidence supporting comments |
|  | 7 | Provide confidential comments to editors |
|  | 8 | Treat author's manuscript with respect, fairness and impartiality |
|  | 9 | Be familiar with the instructions to authors, types of papers, style and standard of the journal you are reviewing for |
|  | 10 | Declare your conflict of interest |
|  | 11 | Maintain confidentiality concerning the existence and contents of unpublished manuscripts |
|  | 12 | Do not review if reviewer has a conflict of interest |
|  | 13 | Compile a neat list of recommendations for authors and editors |
|  | 14 | Do not plagiarize |
|  | 15 | Accept manuscripts for reviews only in yours areas of expertise |
|  | 16 | Make recommendations of acceptance, rejection or revision |
|  | 17 | Identify problems or limitations and help rectify them |
|  | 18 | Reduce misinformation  and confusion |
|  | 19 | Improve the quality of manuscript |
|  | 20 | Provide a list of important items that are missing in the manuscript |
|  | 21 | Point out any contradictions within a manuscript |
|  | 22 | Provide specific comments on the strengths and weaknesses of a submission |
|  | 23 | Have expertise relevant to the research methodology |
|  | 24 | Raise arguments about data in a previous publication that were already made through peer review |
|  | 25 | Evaluate the theoretical framework |
|  | 26 | Evaluate the scientific rigor |
|  | 27 | Suggest alternative titles |
|  | 28 | Evaluate if the article is relevant for the journal |
|  | 29 | Protect the integrity of his or her speciality or subspeciality, the reputation of the journal, the welfare of human and animals subjects |
|  | 30 | Assure that the general review quality is constructive knowledge, logical, analytical, clear/transparent, precise, balanced/fair, courteous |
| **Rational** | 31 | Review consideration of the context of previous relevant research, and the appropriateness or need for conducting the particular study |
|  | 32 | Evaluate the quality of literature review and medical context |
|  | 33 | Discuss the importance and originality of study |
|  | 34 | Evaluate the justification for conducting the study |
|  | 35 | Evaluate the clarity of the rationale |
|  | 36 | Evaluate if the logic of the rationale is clear |
|  | 37 | Evaluate if hypotheses are clearly stated |
|  | 38 | Evaluate if the research question is congruent with the hypotheses and appropriately described |
|  | 39 | Evaluate if objectives are clearly described |
|  | 40 | Check that the study is based on evidence based practice of known effectiveness |
|  | 41 | Evaluate the usefulness of the research for patients and clinical practice |
|  | 42 | Check if preliminary studies exist |
|  | 43 | Check if competing studies exist |
|  | 44 | Check if the study provides original data not available in the literature |
|  | 45 | Evaluate the relevance of the study: duration/relevance |
| **Methods** | 46 | Evaluate the type of article: if it complete articulates a theory or conceptual framework |
|  | 47 | Evaluate if there is congruence between the theoretical or conceptual framework and the problem |
|  | 48 | Evaluate the reliability and validity of the outcome measures |
|  | 49 | Evaluate the methodology quality of the study design/Scientific quality and rigor of the method |
|  | 50 | Evaluate if the methods used are valid and reliable |
|  | 51 | Evaluate if the design is congruent and appropriately described |
|  | 52 | Evaluate if the study design is clear |
|  | 53 | Evaluate the adequacy of the design and methods for the objective |
|  | 54 | Evaluate if there are no serious methodological errors |
|  | 55 | Identify strengths and weaknesses of methods |
|  | 56 | Evaluate if authors used all techniques to avoid risk of bias |
|  | 57 | Evaluate if the experimental design includes sufficient controls |
|  | 58 | Evaluate if the methods are sufficiently and clearly detailed so that a reader could hypothetically reproduce the study |
|  | 59 | Evaluate if the author provided rationale for the methodological choices |
|  | 60 | Evaluate if the method maximizes internal and external validity as appropriate for the chosen study design |
|  | 61 | Evaluate if there is no ambiguity or omission regarding materials, procedures, durations, sample sizes and selection method |
|  | 62 | Evaluate if the operational definitions of the variables is clear |
|  | 63 | Evaluate if the literature review is correctly performed |
|  | 64 | Comment on the methods of the research |
|  | 65 | Evaluate if authors use a robust method for disseminating results |
|  | 66 | Evaluate if there is an adequate description of the setting and subjects |
|  | 67 | Evaluate if authors use an appropriate subject population |
|  | 68 | Evaluate if inclusion and exclusion criteria are clear |
|  | 69 | Evaluate the relevance of inclusion criteria |
|  | 70 | Evaluate the relevance of exclusion criteria |
|  | 71 | Evaluate if authors defined stopping rules |
|  | 72 | Evaluate if the sample size is adequate to detect clinically meaningful effects in pre-specified primary outcomes |
|  | 73 | Evaluate if the sample size calculation is reported |
|  | 74 | Evaluate the randomization procedure |
|  | 75 | Evaluate the relevance of randomization methods |
|  | 76 | Verify that authors register his or her study in a clinical trial registry |
|  | 77 | Verify that authors respect their study protocol |
|  | 78 | Check If the study deviates from the protocol, verify authors explanations |
|  | 79 | In case of a control group, evaluate if it is appropriate |
|  | 80 | In case of a control group, evaluate if it is clearly described |
|  | 81 | If authors use a new scale, evaluate if the measurement instruments are completely described |
|  | 82 | If authors use a new scale, evaluate if authors explained in which population the measurement instrument was developed or validated |
|  | 83 | If authors use a new scale, evaluate if authors describe factor analyses used for developing new scales and subscales |
|  | 84 | If authors use a new scale, evaluate construct measurements |
|  | 85 | Evaluate the quality of the description of the management of adverse events |
|  | 86 | Evaluate if subgroups are relevant |
|  | 87 | In case of multiple groups, evaluate if they are statistically comparable |
|  | 88 | In case of a qualitative study, evaluate if the method used to collect data is appropriate to identify qualitative focus and clearly described |
|  | 89 | In case of a qualitative study, evaluate if the author identified the phenomenon of interest |
|  | 90 | In case of a qualitative study, evaluate if the author stated why a qualitative approach was use |
|  | 91 | In case of a qualitative study, evaluate if authors discussed data saturation |
|  | 92 | In case of an intervention, evaluate if it is adequately described |
| **Statistics** | 93 | Evaluate the relevance of statistical analyses |
|  | 94 | Evaluate if statistical methods are appropriate |
|  | 95 | Evaluate if authors justify the choice of statistic method and statistical tests |
|  | 96 | Verify that authors correctly describe the population |
|  | 97 | Evaluate the choice of T0 (initial time measure), duration, frequency, interval used by authors |
|  | 98 | Evaluate which variables are used by authors |
|  | 99 | Evaluate the statistical model, estimators and assumptions |
|  | 100 | Evaluate the parameterization of statistical models |
|  | 101 | Evaluate data presentation |
|  | 102 | Verify that authors provide confidence intervals/ p-values/overall fit |
|  | 103 | Verify if the statistical interpretation is appropriate |
|  | 104 | Evaluate data quality |
|  | 105 | Evaluate if there are statistical errors |
|  | 106 | Check if authors respect intention to treat for data analyses |
|  | 107 | Evaluate if authors calculated statistical power a priori |
|  | 108 | Evaluate if authors used a missing data strategy |
|  | 109 | Evaluate if sensitivity analyses are conducted to evaluate the impact of the missing data assumption on the results |
|  | 110 | Check that the people missing from the analyses are carefully described |
|  | 111 | Check if authors make multiple statistical tests, describe a rational choice regarding adjustment for alpha risk |
|  | 112 | Verify that the primary analysis is in intention to treat |
|  | 113 | Evaluate if authors report an interaction term between the effect and subgroup |
|  | 114 | Evaluate the statistical relevance of subgroup analyses |
|  | 115 | Evaluate if authors describe patient follow up |
| **Figures/Tables** | 116 | Evaluate if graphs and tables are understandable without having to refer to the text |
|  | 117 | Evaluate if tables and figures summarize the data |
|  | 118 | Evaluate if figures/tables make the data more understandable |
|  | 119 | Evaluate if figures and tables illustrate the important features of methods and results |
|  | 120 | Evaluate if figures/tables are justified |
|  | 121 | Evaluate if the size of figures/tables are adequate |
|  | 122 | Evaluate if the number of tables is appropriate |
|  | 123 | Check if legends correctly explain the figures |
|  | 124 | Evaluate if figures and tables can be simplified or condensed |
| **Results** | 125 | Evaluate if presentation of results is clear, concise and organized |
|  | 126 | Check if results included in analyses includes all variables presented in the text |
|  | 127 | Check if results present outcomes for each objective |
|  | 128 | Check for the accuracy of results |
|  | 129 | Check if authors differentiate between differences that are statistically significant and those of clinical interest |
|  | 130 | Evaluate if results are reasonable and expected |
|  | 131 | Evaluate if results support the conclusions drawn |
|  | 132 | Evaluate if authors clearly et systematically announce the study findings |
|  | 133 | Evaluate if results clearly describe the existence of missing data |
|  | 134 | Evaluate if authors correctly interpret statistical analyses |
|  | 135 | Evaluate if results found are not affected by confounding factors |
|  | 136 | In case of therapeutic study, evaluate if authors report all adverse events |
|  | 137 | In case of therapeutic study, evaluate if authors use analytic tests for adverse events |
| **Discussion** | 138 | Evaluate if authors discuss the interpretation of results |
|  | 139 | Evaluate if findings are important (according to who (the authors or the reviewer?)?) |
|  | 140 | Evaluate if authors report a possible Hawthorne effect |
|  | 141 | Evaluate if authors include only individuals who would meet criteria for authorship |
|  | 142 | Evaluate if authors give explanations for ineligible or non-randomized cases |
|  | 143 | Evaluate if study findings are clearly articulated |
|  | 144 | Check that authors state whether their hypotheses were proven or not |
|  | 145 | Evaluate if authors report and comment on all sources of bias and limitations |
|  | 146 | Evaluate if authors compare findings and contrast findings with previous work in this area |
|  | 147 | Evaluate if authors adequately synthesize the observations with those in the literature |
|  | 148 | Evaluate if authors explain any unexpected results |
|  | 149 | Evaluate if authors present plausible alternative explanations for their findings |
|  | 150 | Evaluate if authors report all essential observations arising from the experimental design |
|  | 151 | Evaluate if authors fully disclose sources of financial support |
|  | 152 | Evaluate if the authors report if the findings are linked to the theoretical or conceptual framework for the study |
|  | 153 | Evaluate if authors discuss how the study findings will improve patient care |
|  | 154 | Evaluate if authors reassess (reassess what about the research?) the research |
|  | 155 | Evaluate if the discussion is concise |
|  | 156 | Evaluate if authors provide level of evidence for results |
|  | 157 | Evaluate if authors overlook critical references |
|  | 158 | Evaluate if the presentation of the findings misleadingly convince the reader that the data was rigorously obtained and reported |
|  | 159 | Verify that there is a clear separation between exploratory (post-hoc) hypothesis-testing and primary pre planned hypothesis testing |
|  | 160 | Check if authors only analyze subgroups described in the methods |
|  | 161 | Assess that authors comment on the results of the research |
| **Conclusions** | 162 | Evaluate if conclusions are congruent |
|  | 163 | Evaluate if conclusions are justified by the results found in study |
|  | 164 | Evaluate if conclusions are appropriately described |
|  | 165 | Evaluate if conclusions present new findings |
|  | 166 | Evaluate if conclusions present clearly outlined major points for future research and/or clinical practice |
|  | 167 | Evaluate if authors explore and explicitly state implications of the results |
|  | 168 | Evaluate if authors present appropriate recommendations for future studies |
|  | 169 | Evaluate if authors evaluate if research results are generalizable |
|  | 170 | Evaluate if authors disproportionately interpret results |
|  | 171 | Evaluate if study data permits the authors to claim efficacy |
| **Abstract** | 172 | Evaluate if the abstract reflects the manuscript as a whole, in particular the findings |
|  | 173 | Evaluate if the abstract is complementary to the title |
|  | 174 | Calculate the number of words of the manuscript |
|  | 175 | Check that abstract includes keywords |
|  | 176 | Evaluate the relevance of keywords |
| **Introduction** | 177 | Evaluate the quality of the introduction (length, grammar, style) |
|  | 178 | Check if the introduction presents epidemiological data and definitions |
|  | 179 | Check if authors used definition of main terms |
|  | 180 | Evaluate if the introduction argues the rationale of the current study |
|  | 181 | Check if the introduction presents a literature review on the scientific and medical context |
|  | 182 | Evaluate if the study is based on evidence based practice of known effectiveness |
| **References** | 183 | Evaluate if references are correctly formatted |
|  | 184 | Evaluate if there are sufficient references for the purpose of this manuscript |
|  | 185 | Evaluate if the references included are up to date |
|  | 186 | Evaluate if there are no errors in citations |
|  | 187 | Check if authors correctly use references' results |
| **Reporting guidelines** | 188 | Evaluate if authors used reporting guidelines |
|  | 189 | Evaluate if the reporting guidelines are appropriate for the study |
|  | 190 | Evaluate the quality of reporting guidelines |
|  | 191 | Evaluate if there are errors in data reporting |
| **Ethical review** | 192 | Evaluate if the study received ethics review board approval |
|  | 193 | If study did not receive ethics review board approval, evaluate the potential risks and benefits for participants, and the adequacy of the information to be provided to participants |
|  | 194 | If the study did not receive ethics review board approval, evaluate the adequacy of the information provided to participants |
|  | 195 | If the study did not receive ethics review board approval, evaluate the description of patient consent forms |
|  | 196 | If the study did not receive ethics review board approval, evaluate the if there is a discussion regarding the rights of the study’s participants |
|  | 197 | If the study did not receive ethics review board approval, evaluate the research violations about humans or animals |
|  | 198 | If study did not receive ethics review board approval, evaluate if this study respects the Helsinki' Declaration |
| **Fraude** | 199 | Assess the possibility of fraud |
|  | 200 | Investigate for possible plagiarism or duplicate submission |
|  | 201 | Report suspected authorship to editors |
|  | 202 | Evaluate if authors have any conflict of Interest |
|  | 203 | Check if results have been published previously |
|  | 204 | Check if the manuscript has been previously published |
|  | 205 | Evaluate the description of resource allocation |
