# Supplementary figures and images for: The most important tasks for peer reviewers evaluating a randomized controlled trial are not congruent with the tasks most often requested by journal editors
Source: BMC Med. 2015 Jul 3;13:158. doi: 10.1186/s12916-015-0395-3 (PMC4491236; doi:10.1186/s12916-015-0395-3)

Additional file 3. Example of sorting with Qsort


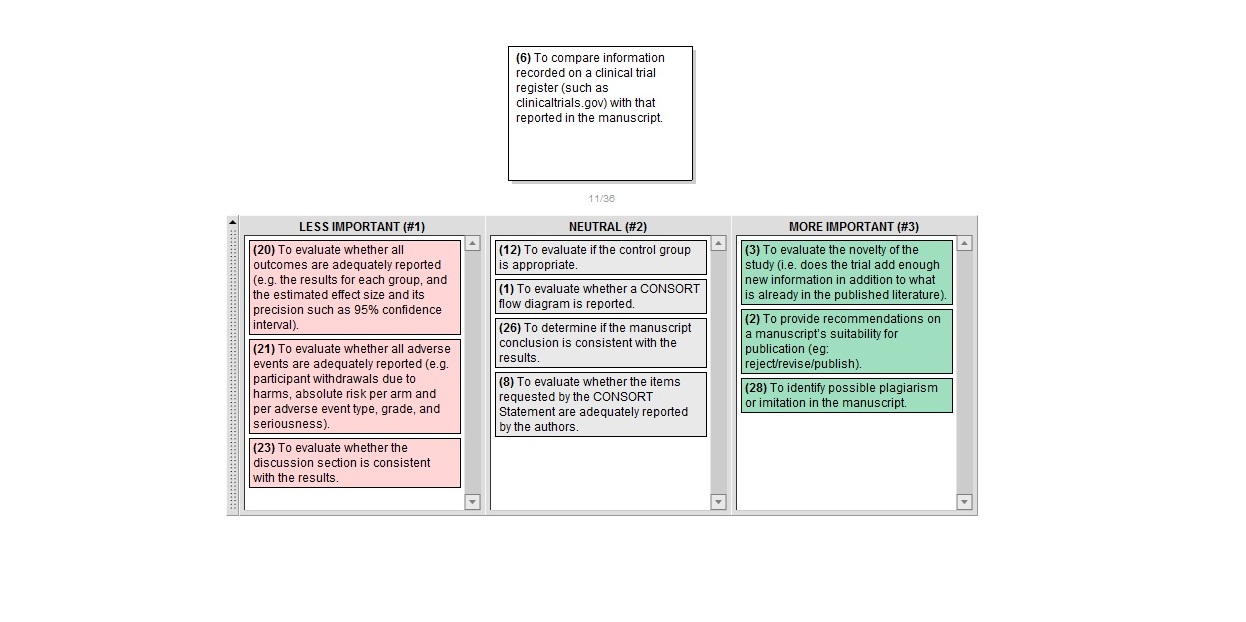


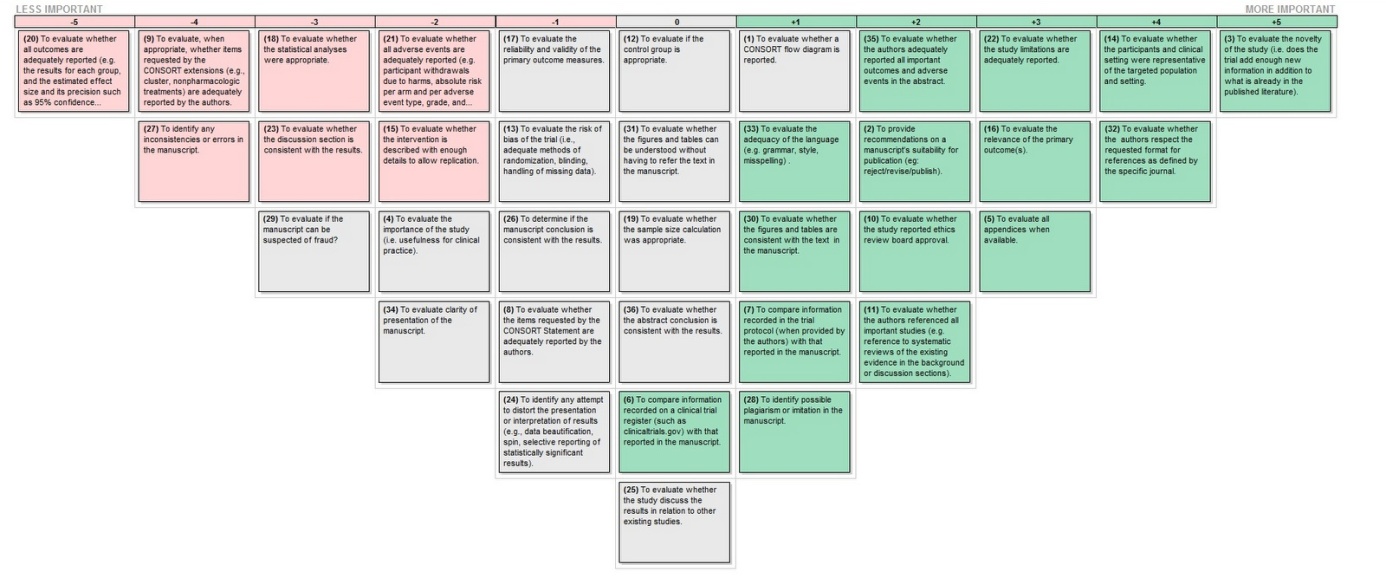

Supplement: Additional file 3: — Example of sorting with Q-sort. [file 12916_2015_395_MOESM3_ESM.doc]
