# Supplementary material for: The most important tasks for peer reviewers evaluating a randomized controlled trial are not congruent with the tasks most often requested by journal editors
Source: BMC Med. 2015 Jul 3;13:158. doi: 10.1186/s12916-015-0395-3 (PMC4491236; doi:10.1186/s12916-015-0395-3)
Supplement: Additional file 4: — Sensitivity analysis of task ranking with and without the editor panel. [file 12916_2015_395_MOESM4_ESM.doc]

Additional file 4. Sensitivity analysis of tasks ranked with and without the editor panel.

| **Tasks** | **Tasks ranking with editors panel**  **N=203** | **Tasks ranking without editors panel**  **N=182** |
| --- | --- | --- |
| To evaluate the risk of bias of the trial | 1 | 1 |
| To determine if the manuscript conclusion is consistent with the results | 2 | 2 |
| To evaluate the adequacy of statistical analyses | 3 | 3 |
| To evaluate if the control group is appropriate | 4 | 5 |
| To check if all outcomes are adequately reported | 5 | 4 |
| To evaluate the relevance of the primary outcome(s) | 6 | 6 |
| To search for any attempt to distort the presentation or interpretation of results | 7 | 7 |
| To evaluate the reliability and validity of the outcome measures | 8 | 8 |
| To evaluate the importance of the study | 9 | 12 |
| To evaluate if the abstract conclusion is consistent with the results | 10 | 10 |
| To evaluate if the discussion is consistent with the results | 11 | 9 |
| To check if all adverse events are adequately reported | 12 | 11 |
| To check if the intervention is described with enough details to allow replication | 13 | 13 |
| To check that limitations are adequately reported | 14 | 14 |
| To evaluate the adequacy of the selection of participants and clinical setting | 15 | 15 |
| To search for any inconsistencies or errors in the manuscript | 16 | 16 |
| To evaluate the novelty of the study | 17 | 20 |
| To check the sample size calculation | 18 | 19 |
| To check if the authors reported all important outcomes and adverse events in the abstract | 19 | 17 |
| To discuss the results in relation to other studies | 20 | 18 |
| To evaluate if the manuscript can be suspected of fraud | 21 | 21 |
| To provide recommendations on publication | 22 | 22 |
| To check if all figures and tables are consistent with the text | 23 | 23 |
| To evaluate clarity of presentation | 24 | 24 |
| To check if the study reported ethics review board approval | 25 | 25 |
| To search for plagiarism or imitation in the paper | 26 | 26 |
| To compare information recorded in the trial protocol when provided by the authors and reported in the manuscript | 27 | 27 |
| To check if the items requested by the CONSORT Statement are adequately reported by authors | 28 | 28 |
| To check if the authors referenced all important studies | 29 | 30 |
| To evaluate whether figures and tables can be understand without having to refer the text | 30 | 29 |
| To check if items requested by the CONSORT extensions are adequately reported when appropriate | 31 | 31 |
| To compare information recorded on clinical trial register such as clinicaltrials.gov and reported in the manuscript | 32 | 32 |
| To read the journals’ recommendations to reviewers | 33 | 33 |
| To evaluate all appendices when available | 34 | 34 |
| To evaluate the adequacy of the language | 35 | 35 |
| To evaluate if authors respect the requested format for references | 36 | 36 |
