# Supplementary material for: The most important tasks for peer reviewers evaluating a randomized controlled trial are not congruent with the tasks most often requested by journal editors
Source: BMC Med. 2015 Jul 3;13:158. doi: 10.1186/s12916-015-0395-3 (PMC4491236; doi:10.1186/s12916-015-0395-3)
Supplement: Additional file 5: — Articles selected during the literature review. [file 12916_2015_395_MOESM5_ESM.doc]

Additional file 5. Articles selected during the literature review

**Articles with specific recommendations in the article which were extracted**:

1. Alam, M., et al., Blinded vs. unblinded peer review of manuscripts submitted to a dermatology journal: a randomized multi-rater study. Br J Dermatol, 2011. 165(3): p. 563-7.
2. Albers, C.A., et al., Publication criteria and recommended areas of improvement within school psychology journals as reported by editors, journal board members, and manuscript authors. J Sch Psychol, 2011. 49(6): p. 669-89.
3. Ammenwerth, E., et al., Developing and evaluating criteria to help reviewers of biomedical informatics manuscripts. J Am Med Inform Assoc, 2003. 10(5): p. 512-4.
4. Azer, S.A., S. Ramani, and R. Peterson, Becoming a peer reviewer to medical education journals. Med Teach, 2012. 34(9): p. 698-704.
5. Bayne, S.C., G.P. McGivney, and S.C. Mazer, Scientific composition and review of manuscripts for publication in peer-reviewed dental journals. J Prosthet Dent, 2003. 89(2): p. 201-18.
6. Benos, D.J., et al., The ups and downs of peer review. Adv Physiol Educ, 2007. 31(2): p. 145-52.
7. Benos, D.J., K.L. Kirk, and J.E. Hall, How to review a paper. Adv Physiol Educ, 2003. 27(1-4): p. 47-52.
8. Broome, M., et al., Ethical concerns of nursing reviewers: an international survey. Nurs Ethics, 2010. 17(6): p. 741-8.
9. Bydder, S., et al., Assessment of abstracts submitted to the annual scientific meeting of the Royal Australian and New Zealand College of Radiologists. Australas Radiol, 2006. 50(4): p. 355-9.
10. Christenbery, T.L., Manuscript peer review: a guide for advanced practice nurses. J Am Acad Nurse Pract, 2011. 23(1): p. 15-22.
11. Einarson, T.R. and G. Koren, To accept or reject? A guide to peer reviewing of medical journal papers. J Popul Ther Clin Pharmacol, 2012. 19(2): p. e328-33.
12. Gasparyan, A.Y., et al., Writing a narrative biomedical review: considerations for authors, peer reviewers, and editors. Rheumatol Int, 2011. 31(11): p. 1409-17.
13. Gasparyan, A.Y., L. Ayvazyan, and G.D. Kitas, Authorship problems in scholarly journals: considerations for authors, peer reviewers and editors. Rheumatol Int, 2013. 33(2): p. 277-84.
14. Gupta, P., et al., What is submitted and what gets accepted in Indian Pediatrics: analysis of submissions, review process, decision making, and criteria for rejection. Indian Pediatr, 2006. 43(6): p. 479-89.
15. Harris, A., R. Reeder, and J. Hyun, Survey of editors and reviewers of high-impact psychology journals: statistical and research design problems in submitted manuscripts. J Psychol, 2011. 145(3): p. 195-209.
16. Harris, A.H., R. Reeder, and J.K. Hyun, Common statistical and research design problems in manuscripts submitted to high-impact psychiatry journals: what editors and reviewers want authors to know. J Psychiatr Res, 2009. 43(15): p. 1231-4.
17. Henly, S.J. and M.C. Dougherty, Quality of manuscript reviews in nursing research. Nurs Outlook, 2009. 57(1): p. 18-26.
18. Houry, D., S. Green, and M. Callaham, Does mentoring new peer reviewers improve review quality? A randomized trial. BMC Med Educ, 2012. 12: p. 83.
19. Kliewer, M.A., et al., Reviewing the reviewers: comparison of review quality and reviewer characteristics at the American Journal of Roentgenology. AJR Am J Roentgenol, 2005. 184(6): p. 1731-5.
20. Landkroon, A.P., et al., Quality assessment of reviewers' reports using a simple instrument. Obstet Gynecol, 2006. 108(4): p. 979-85.
21. Landy, D.C., et al., Prepublication review of medical ethics research: cause for concern. Acad Med, 2009. 84(4): p. 495-7.
22. Lee, K.P., et al., Predictors of publication: characteristics of submitted manuscripts associated with acceptance at major biomedical journals. Med J Aust, 2006. 184(12): p. 621-6.
23. Levine, A.M., J.D. Heckman, and R.N. Hensinger, The art and science of reviewing manuscripts for orthopaedic journals: Part I. Defining the review. Instr Course Lect, 2004. 53: p. 679-88.
24. Lippert, S., M.L. Callaham, and B. Lo, Perceptions of conflict of interest disclosures among peer reviewers. PLoS One, 2011. 6(11): p. e26900.
25. Lovejoy, T.I., T.A. Revenson, and C.R. France, Reviewing manuscripts for peer-review journals: a primer for novice and seasoned reviewers. Ann Behav Med, 2011. 42(1): p. 1-13.
26. McLeod, P., et al., Peer review: an effective approach to cultivating lecturing virtuosity. Med Teach, 2013. 35(4): p. e1046-51.
27. Peh, W.C. and K.H. Ng, Role of the manuscript reviewer. Singapore Med J, 2009. 50(10): p. 931-3; quiz 934.
28. Rivara, F.P., et al., A comparison of reviewers selected by editors and reviewers suggested by authors. J Pediatr, 2007. 151(2): p. 202-5.
29. Rowe, B.H., et al., Reviewer agreement trends from four years of electronic submissions of conference abstract. BMC Med Res Methodol, 2006. 6: p. 14.
30. Rutkowski, J.L. and J.V. Cairone, How to review scientific manuscripts and clinical case reports for Journal of Oral Implantology. J Oral Implantol, 2009. 35(6): p. 310-4.
31. Shattell, M.M., et al., Authors' and editors' perspectives on peer review quality in three scholarly nursing journals. J Nurs Scholarsh, 2010. 42(1): p. 58-65.
32. Triggle, C.R. and D.J. Triggle, What is the future of peer review? Why is there fraud in science? Is plagiarism out of control? Why do scientists do bad things? Is it all a case of: "all that is necessary for the triumph of evil is that good men do nothing"? Vasc Health Risk Manag, 2007. 3(1): p. 39-53.
33. Wager, E., E.C. Parkin, and P.S. Tamber, Are reviewers suggested by authors as good as those chosen by editors? Results of a rater-blinded, retrospective study. BMC Med, 2006. 4: p. 13.
34. Yaffe, M.B., Re-reviewing peer review. Sci Signal, 2009. 2(85): p. eg11.

**Articles subsequently found to include no relevant data and were excluded:**

1. Abdoul, H., et al., Peer review of grant applications: criteria used and qualitative study of reviewer practices. PLoS One, 2012. 7(9): p. e46054.
2. Anraku, A., et al., Survey of conflict-of-interest disclosure policies of ophthalmology journals. Ophthalmology, 2009. 116(6): p. 1093-6.
3. Baggs, J.G., et al., Blinding in peer review: the preferences of reviewers for nursing journals. J Adv Nurs, 2008. 64(2): p. 131-8.
4. Borgwardt, S., et al., Why are psychiatric imaging methods clinically unreliable? Conclusions and practical guidelines for authors, editors and reviewers. Behav Brain Funct, 2012. 8: p. 46.
5. Bornmann, L., R. Mutz, and H.D. Daniel, A reliability-generalization study of journal peer reviews: a multilevel meta-analysis of inter-rater reliability and its determinants. PLoS One, 2010. 5(12): p. e14331.
6. Brand, R.A., Reviewing for clinical orthopaedics and related research. Clin Orthop Relat Res, 2012. 470(9): p. 2622-5.
7. Callaham, M.L. and J. Tercier, The relationship of previous training and experience of journal peer reviewers to subsequent review quality. PLoS Med, 2007. 4(1): p. e40.
8. Clark, R.K., Peer review: a view based on recent experience as an author and reviewer. Br Dent J, 2012. 213(4): p. 153-4.
9. Costantino, G., et al., Errors in medical literature: not a question of impact. Intern Emerg Med, 2013. 8(2): p. 157-60.
10. Davidhizar, R. and G.A. Bechtel, Tips for manuscript reviewers. Nurse Author Ed, 2003. 13(3): p. 1-4.
11. Dawson, A.J. and S.M. Yentis, Contesting the science/ethics distinction in the review of clinical research. J Med Ethics, 2007. 33(3): p. 165-7.
12. De Jong, M.J., Spotting reference errors. Nurse Author Ed, 2004. 14(2): p. 7-9.
13. Duff, K., et al., On becoming a peer reviewer for a neuropsychology journal. Arch Clin Neuropsychol, 2009. 24(3): p. 201-7.
14. Emerson, G.B., et al., Testing for the presence of positive-outcome bias in peer review: a randomized controlled trial. Arch Intern Med, 2010. 170(21): p. 1934-9.
15. Forster, A.J., et al., Reliability of the peer-review process for adverse event rating. PLoS One, 2012. 7(7): p. e41239.
16. Gasparyan, A.Y. and G.D. Kitas, Best peer reviewers and the quality of peer review in biomedical journals. Croat Med J, 2012. 53(4): p. 386-9.
17. Gollogly, L. and H. Momen, Ethical dilemmas in scientific publication: pitfalls and solutions for editors. Rev Saude Publica, 2006. 40 Spec no.: p. 24-9.
18. Grainger, D.W., Peer review as professional responsibility: a quality control system only as good as the participants. Biomaterials, 2007. 28(34): p. 5199-203.
19. Hall, P.A., R. Poulsom, and J. Wixon, How does The Journal of Pathology deal with conflict of interest? J Pathol, 2009. 219(4): p. 396-9.
20. Harden, R.M. and P. Lilley, A fresh approach to publishing and reviewing papers in health professions education. Med Teach, 2013. 35(1): p. 1-3.
21. Henly, S.J., J.A. Bennett, and M.C. Dougherty, Scientific and statistical reviews of manuscripts submitted to Nursing Research: Comparison of completeness, quality, and usefulness. Nurs Outlook, 2010. 58(4): p. 188-99.
22. Herbison, P., How to make your article more acceptable for the statistical reviewer. Neurourol Urodyn, 2007. 26(3): p. 318-22.
23. Hrynaszkiewicz, I., et al., Preparing raw clinical data for publication: guidance for journal editors, authors, and peer reviewers. Trials, 2010. 11: p. 9.
24. Jacobson, A.F., K. Schmidt, and H. Coeling, Preserving blind peer review of electronic manuscript files. Nurse Author Ed, 2005. 15(1): p. 1-4, 7.
25. Johnston, S.C., et al., Early editorial manuscript screening versus obligate peer review: a randomized trial. Ann Neurol, 2007. 61(4): p. A10-2.
26. Jones, A.W., The distribution of forensic journals, reflections on authorship practices, peer-review and role of the impact factor. Forensic Sci Int, 2007. 165(2-3): p. 115-28.
27. Kadar, N., Systemic bias in peer review: suggested causes, potential remedies. J Laparoendosc Adv Surg Tech A, 2010. 20(2): p. 123-8.
28. Kearney, M.H. and M.C. Freda, Nurse editors' views on the peer review process. Res Nurs Health, 2005. 28(6): p. 444-52.
29. Leek, J.T., M.A. Taub, and F.J. Pineda, Cooperation between referees and authors increases peer review accuracy. PLoS One, 2011. 6(11): p. e26895.
30. Levine, A.M., J.D. Heckman, and R.N. Hensinger, The art and science of reviewing manuscripts for orthopaedic journals: Part II. Optimizing the manuscript: practical hints for improving the quality of reviews. Instr Course Lect, 2004. 53: p. 689-97.
31. Loonen, M.P., J.J. Hage, and M. Kon, Who benefits from peer review? An analysis of the outcome of 100 requests for review by Plastic and Reconstructive Surgery. Plast Reconstr Surg, 2005. 116(5): p. 1461-72; discussion 1473-5.
32. Ludbrook, J., Peer review of biomedical manuscripts: an update. J Clin Neurosci, 2003. 10(5): p. 540-2
33. Morton, J.P., Reviewing scientific manuscripts: how much statistical knowledge should a reviewer really know? Adv Physiol Educ, 2009. 33(1): p. 7-9.
34. Nahata, M.C., Tips for writing and publishing an article. Ann Pharmacother, 2008. 42(2): p. 273-7.
35. Newton, D.P., Quality and peer review of research: an adjudicating role for editors. Account Res, 2010. 17(3): p. 130-45.
36. Pierson, D.J., The top 10 reasons why manuscripts are not accepted for publication. Respir Care, 2004. 49(10): p. 1246-52.
37. Provenzale, J.M. and R.J. Stanley, A systematic guide to reviewing a manuscript. AJR Am J Roentgenol, 2005. 185(4): p. 848-54.
38. Rajesh, A., G. Cloud, and M.G. Harisinghani, Improving the quality of manuscript reviews: impact of introducing a structured electronic template to submit reviews. AJR Am J Roentgenol, 2013. 200(1): p. 20-3.
39. Ramulu, V.G., et al., Development of a case report review instrument. Int J Clin Pract, 2005. 59(4): p. 457-61.
40. Rangachari, P.K., Teaching undergraduates the process of peer review: learning by doing. Adv Physiol Educ, 2010. 34(3): p. 137-44.
41. Sosa, J.A., et al., Evaluating the surgery literature: can standardizing peer-review today predict manuscript impact tomorrow? Ann Surg, 2009. 250(1): p. 152-8.80
42. Steinhauser, G., et al., Peer review versus editorial review and their role in innovative science. Theor Med Bioeth, 2012. 33(5): p. 359-76.
43. Timmer, A., L.R. Sutherland, and R.J. Hilsden, Development and evaluation of a quality score for abstracts. BMC Med Res Methodol, 2003. 3: p. 2.
44. Tite, L. and S. Schroter, Why do peer reviewers decline to review? A survey. J Epidemiol Community Health, 2007. 61(1): p. 9-12.
45. Vinther, S., et al., Same review quality in open versus blinded peer review in "Ugeskrift for Laeger". Dan Med J, 2012. 59(8): p. A4479.
46. Wagner, A.K., et al., Peer review: issues in physical medicine and rehabilitation. Am J Phys Med Rehabil, 2003. 82(10): p. 790-802.
47. Williams, E.H., P.A. Carpentier, and T. Misteli, Minimizing the "re" in review. J Cell Biol, 2012. 197(3): p. 345-6.
48. Wong, V.S., The examination of peer review and publication in neurology. J Child Neurol, 2010. 25(10): p. 1298-301.

**Articles referred to other sources information from which further data was extracted (such as online training resources, websites etc):**

1. Curzon, M.E. and P.E. Cleaton-Jones, Reviewing scientific manuscripts. Eur Arch Paediatr Dent, 2011. 12(4): p. 184-7.
2. Eccles, M.P., et al., Implementation Science six years on--our evolving scope and common reasons for rejection without review. Implement Sci, 2012. 7: p. 71.
3. Lane, J.A., et al., A Peer Review Intervention for Monitoring and Evaluating sites (PRIME) that improved randomized controlled trial conduct and performance. J Clin Epidemiol, 2011. 64(6): p. 628-36.
4. Mazumdar, M., S. Banerjee, and H.L. Van Epps, Improved reporting of statistical design and analysis: guidelines, education, and editorial policies. Methods Mol Biol, 2010. 620: p. 563-98.
5. Nelson, C.A., S.R. Freeman, and R.P. Dellavalle, Reviewing dermatology manuscripts and publications. Dermatol Clin, 2009. 27(2): p. 201-4, viii.
6. Schroter, S., et al., Effects of training on quality of peer review: randomised controlled trial. BMJ, 2004. 328(7441): p. 673.
7. Schroter, S., et al., What errors do peer reviewers detect, and does training improve their ability to detect them? J R Soc Med, 2008. 101(10): p. 507-14.
8. Snell, L. and J. Spencer, Reviewers' perceptions of the peer review process for a medical education journal. Med Educ, 2005. 39(1): p. 90-7
